# Using Speculative Fiction to Imagine Queer Abolition Real Utopias

**DOI:** 10.1007/s10612-024-09793-1

**Published:** 2024-07-29

**Authors:** Kayleigh Charlton

**Affiliations:** University of Bath, Bath, UK

## Abstract

This article uses speculative fiction as a method for exploring the potentialities of queer abolition utopias. Abolition utopias aim to strike a balance of hope and need, offering innovative alternatives to prison while also addressing the current penal and social realities of marginalised groups. Queer abolition utopias, informed by the literature in queer criminology, centres the experiences of LGBTQ + people in these innovative alternatives. This article presents 3 pieces of short speculative fiction (1) Finding Harmony House, (2) Glasgow East Community Forum, (3) Our Long weekend at Leuchars Lodge. All 3 stories centre an LGBTQ + character(s) who are facing a particular challenge or challenges in their life, and how said alternatives might play a role in their moving forward.

## Introduction

Underpinned by a queer criminological analysis, this article builds on the work of David Scott’s (2013) abolition real utopias. Abolition real utopias aim to strike a balance of hope and need, offering both fruitful and innovative alternatives to prison while also addressing current penal realities. Scott’s work thus far has outlined an abolitionist compass to help us conceptualise abolition real utopias and has offered a range of alternatives itself. Abolition real utopias, however, will look different for every community; Radical Alternatives to Prison (1971, as cited in Scott, 2013) suggests “there can be no blanket alternative to prison; only a series of different schemes for all the different offenders” (p. 14). This work bridges the literature on abolition real utopias with queer criminology, a nascent subfield of criminology that seeks to engage with LGBTQ + issues, through the use of speculative fiction writing as a method. This article’s aim is twofold; it seeks to both conceptualise what queer specific abolition real utopias might look like and secondly to explore what speculative fiction as a method can offer criminological inquiry. Queer criminology has arguably fallen short of its radical potential thus far by not centring abolition. This article offers new directions for queer criminology and encourages those interested in LGBTQ + lives and experiences in the carceral system to think beyond the current institutions we have in front of us.

This article offers a glimpse into queer abolition real utopias through a series of flash fiction pieces. Speculative fiction can help foster hope; it offers a way for us to imagine solutions and alternative futures beyond what currently exists. In this case, speculative fiction is used to offer a way for us to distance our minds from current criminal justice responses and imagine new, just responses to the social and penal realities of today. Indeed, prison and the surrounding current criminal justice system has become so normalised that it is difficult to imagine a world without them (Davis, 2003). Subsequently, attempts at imagining alternatives are stifled. Importantly, then, using speculative fiction to theorise can help illustrate what is possible beyond this realm (Gerlach and Hamilton, 2003). There are 3 short stories in this article, all of which centre an LGBTQ character(s) and explore—in brief—an alternative to the current criminal justice system. These alternatives are not necessarily fully formed, or in-depth, but they are used to firstly demonstrate the endless possibilities of alternatives and secondly, the way in which speculative fiction can help us move in new directions.

This article begins with an overview of the literature on abolition utopias and queer abolition politics, introducing David Scott’s (2013) work on abolition utopias followed by some discussion about feminist and queer criminology, including some of the critiques surrounding queer criminology so far. Then, the article explores why we need queer specific abolition utopias by examining the current penal realities of LGBTQ + people in more detail, as well as the needs and experiences of the wider LGBTQ + population. The article then touches on the role of speculative fiction as a method for imagining new futures, and its connection to abolitionist writing. At this point, the article introduces the series of fiction pieces, weaving in an academic commentary that guides the creative directions the author has taken in the writing. The article then offers concluding comments on the potentialities of queer abolition real utopias and the future direction of queer criminology; specifically, that queer criminology should be engaging in more radical and innovative work and speculative fiction is just one of the ways in which we can encourage this.

## ‘Real Utopias’, Abolition Utopias and Queer Abolition Politics

### Defining ‘Real Utopias’

A ‘real utopia’ merges both a utopia and an achievable goal. Real utopias represent a hopeful future—one that both challenges existing social structures and is just. This concept lies in contrast to a traditional utopia, given that they are often considered unattainable or idealistic. The term was used by Erik Olin Wright in *Envisioning Real Utopias* (2010) where he wrote about bridging the gap between an abstract utopian ideal and a pragmatic reform. Real utopias, then, serve as an opportunity to reframe and reconsider how we can bring a traditional utopia to life through practical policies or interventions. Some of the key features we might associate with a real utopia, as opposed to a traditional one, is feasibility and viability. Importantly, real utopias can be used in a wide range of ways, from criticising existing dominant systems to inspiring change. As a framework, real utopias can help us not only imagine a better, more just society, but offer practical steps or strategies to get us there.

### Abolition Real Utopias

*“*To speak metaphorically, in shark infested waters people are not likely to jump ship no matter how obvious it is that it is sinking unless they have something to jump onto. Without rational alternatives unjust penal and social systems will appear permanent and inevitable.” (Scott, 2013, p. 97).

In 1990, Stanley Cohen called for penal abolitionists to focus on pragmatic interventions that also incorporate an element of utopianism of how current realities can be transcended. Since then, multiple scholars have attempted to strike the somewhat contradictory demands of Cohen’s call (Giddens, 1994; Wright, 2010), including David Scott’s (2013) work on abolition real utopias. Scott argues that an abolitionist real utopia is made up of three central components: (1) it diagnoses and critiques the power to punish, (2) it advocates for visions of radical alternatives grounded in progressive normative principles and (3) it has a clear strategy of emancipatory change that seeks to build new realities outside of the capitalist state. Further, Scott discusses what he coins ‘an abolitionist compass’ which helps us balance the need for said immediate humanitarian aid while not legitimising the existing systems. This compass is used, then, to visualise ‘real’ utopia interventions; or in other words, utopian, ambitious, new worldly visions alongside immediate interventions to ease the suffering of people impacted by the criminal justice system. These interventions combine both immediate direct policy interventions alongside community-based social movements and are otherwise in opposition to both criminal justice reform and utopian ideology that fails to account for current penal realities, for example, interventions such as gender-informed care or LGBTQ-informed care that merely seek to reform existing interventions to meet the needs of marginalised groups. While these reforms may be well-intended, these practices simply sustain and expand the carceral system rather than offering alternatives to imprisonment.

Scott offers a range of possible options rather than pre-determined solutions that adhere to the principles of abolition real utopias, citing Mathieson who wrote that “the finished alternative is finished in the double sense of the word” (1974, p. 13). Scott rather focusses his attention on the abolition compass. The compass is underscored by six guiding principles that can aid us in understanding what a utopian ‘real’ intervention might look like. These principles include the protection of human dignity and minimising of human suffering, the emancipatory values of social justice, the logic of the ‘competing contradiction’, the promise of a genuine alternative to the criminal process, and the incorporation of legal safeguards and interventions that are relevant, meaningful and allow for constructive participation (Scott, 2013). This article looks to build on Scott’s (2013) ideas thus far by highlighting the possibilities of queer ‘real’ utopian interventions that are underpinned by a queer criminological analysis. Queer politics and queer criminology together could offer a fruitful analysis of what abolition real utopias that are specific to the issues that queer communities face might look like.

### Feminist and Queer Criminology

There is a long history of queer people resisting the police and the criminal justice system more broadly; indeed, these tactics and visions have been integral to queer organising for decades and they continue to inform queer liberation today. However, scholarship on LGBTQ + resistance and populations in criminological research was thin until the 1990s. It was not until the early 2010s that Queer Criminology became formally recognised at the likes of the American Society of Criminology annual meetings. The panels, in turn, led to the publication of the first collection of scholarly works that formed the foundations of queer criminology, *The Handbook of LGBT Communities, Crime and Justice* (Peterson and Panfill, 2013). Since then, queer criminology has garnered the attention of researchers across the globe, with work on a range of methodological, theoretical, and pedagogical concerns.

Walker et al., (2022) argue, however, that the bulk of the work being done in this area is largely reformist in nature. Further, according to zemiologists, queer criminology thus far has stopped shy of its radical potential by not centring abolitionism and failing to offer a “fundamental reimagining of criminology as an enterprise in itself” (Copson and Boukli, 2020, p. 513). In other words, queer criminology has been criticised for attempting to improve existing frameworks and the experiences of LGBTQ + people in the criminal justice system, rather than challenging the system more widely. While this has been the case with some queer criminology, there is a growing body of work that examines abolitionist alternatives to traditional criminalising processes (Stanley, 2011; Hobson, 2016; Rodriguez, 2021). Moreover, there is a plethora of research within feminist criminology that explores abolition alternatives through the lens of gender inequality that this work can draw on (Russel and Carlton 2013; Kim, 2018; Lamusse, 2022).

There has been an extensive amount written from an anti-carceral feminist perspective (Taylor, 2018; Terwiel, 2019; D’Avolio et al., 2023). This branch of feminism specifically warns of the dangers of additional legislation and increased sentencing as a response to gender-based violence. This work has also been seen in activist spaces, with groups like INCITE! and Chrysalis Collective producing approaches for dealing with violence without the involvement of the criminal legal justice system. INCITE! formed to develop strategies to simultaneously end violence from both the state and the community. They centre political analysis and community action in their struggle for liberation, recognising that state violence and community violence disproportionality impact and punish women, trans people, and gender non-conforming people of colour.

So, while there is a diverse body of literature and an ongoing commitment to activism that is rooted in feminist and queer approaches to abolition, this has not yet been centred by queer criminologists. Thus, there remains limited work within queer criminology that adopts a broader understanding of the notion of “queer” that moves beyond identity categories and centres the terms disruptive possibilities (Ball, 2016). There is scope for queer criminology to engage and indeed pioneer queer ‘real’ utopia interventions. A queer abolition utopia would not only centre the demands of ‘real’ utopias but focus specifically on interventions, immediate and long-term, that are specific to the struggles faced by the LGBTQ + community.

## Specific Needs and Experiences of At-Risk LGBTQ + Populations

It is well established that LGBTQ + people experience higher rates of poor mental health than the general population, with higher rates of eating problems, suicidality, self-harm, and addiction (Jorm et al., 2001; Colledge et al., 2015). The transgender community faces even greater risk of suicide, depression, substance use and general distress (Donohue et al., 2021). In a recent government equalities office (2018), LGBTQ + people responded that they were less satisfied with their life compared to the general UK population. At least two thirds of LGBTQ+ respondents experienced verbal harassment or physical violence in the last 12 months because of their identity. The survey also revealed that trans individuals were less likely to be employed than the UK general population, and that there was an increased risk of homelessness among LGBTQ + youth. This is echoed by Stonewall (2018), who found that 1 in 4 trans people had been discriminated against when seeking rented accommodation. This, in turn, puts the LGBTQ + community at greater risk of engagement with the criminal justice system.

LGBTQ + populations have a long history of being heavily criminalised and remain consistently over-represented in the criminal justice system. While only 3% of the general population in the UK identify as LGBTQ + , more than double the prison population identify within this category with 7% identifying as LGBTQ + in men’s prisons, and 22% in women’s prisons (Prison Reform Trust, 2021). Indeed, in prison, the already disproportionate challenges faced by LGBTQ + people are often compounded by institutional stigma, alienation, and victimisation (Rodgers et al., 2017; Donohue et al., 2021; Walters et al., 2024). While there is limited research on LGBTQ + experiences in prisons—in the UK, at least—there are some common themes across the literature. This includes a lack of LGBTQ + specific support, concerns about disclosing one’s LGBTQ + identity and institutional discrimination (Fernandes et al., 2020). Furthermore, Carr et al., (2016) argue that while homophobia and transphobia in women’s prisons may appear to be less of a problem, this is not necessarily the case, with reports of discrimination, abuse and violence faced by lesbian, bisexual, and transgender women in prison. The trans community in prison face additional barriers, including heightened isolation, transphobia and challenges associated with transitioning (Maycock, 2022). Beyond the UK, there is a growing body of work from scholars in the US on the experiences of transgender prisoners (Velasquez-Potts, 2021; Vogler and Rosales, 2022). These articles reveal the marginalisation and the unique forms of gendered violence faced by gender non-conforming and transgender prisoners.

These unique challenges faced by the LGBTQ + community, particularly those considered ‘at-risk’, demonstrate a need for LGBTQ + specific real utopias. This article chooses to focus on access to safe spaces, handling conflict without criminal law and voluntary and family support. Indeed, these are just a few of the possible routes to support the LGBTQ + community without the need for the current criminal justice system. Of course, these options are not an exhaustive list of alternative options to explore, and by no means are the alternatives paths *alone* enough for radical change but they begin to open our mind to difference, to change and ultimately, to a new future.

## Speculative Fiction as a Method

Speculative fiction is considered both an abolitionist tool (Kãrklina, 2021) and a tool of critical pedagogy (Houlden and Veletsianos, 2023). Speculative fiction has been used for decades to imagine new worlds, described as the “perfect testing ground” for abolitionist ideas. This is particularly true today, where police and prisons are seen as integral parts of our world and our criminal justice system. Fiction of this kind is reflective, analytical, and critical and can be used to envision different, more egalitarian worlds and ways of connecting. Indeed, speculative fiction has been used throughout history by numerous writers to look to the future and imagine new, just worlds. Both Octavia Butler (1993) and Le Guin (1974) are among the most renowned for their work in the genre of speculative fiction, built upon years of research about the likes of anarchism, capitalist exploitation, and mutual aid. While many novels, films and television shows have followed in the footsteps of these authors, speculative fiction remains overlooked in academic spaces (Thorne, 2021).

Fiction is not only a way to explore new ideas in academic writing, but it is inherently more engaging and accessible to wider audiences than traditional academic writing. This has resulted in some recent push-back, with writers such as Kitchin (2013**)** arguing for more engagement with non-traditional writing forms in academia. This has been echoed not only in the humanities and social sciences but across STEM subjects too, science communication having encouraged the use of storytelling to make “science comprehensible and meaningful for general audiences” (ElShafie, 2018, p. 1213). There is a call for sociologists and social scientists in particular to engage in more interesting writing forms (Rinehart, 1998; Richardson, 2000). Fiction is just one of the many forms we can choose; Gerlach and Hamilton (2003) highlight the pre-existing connection between the social sciences and science fiction—indeed, both examine social problems and consider visions of a better future. However, Greenberg and Warrick (1974) argued that while politics and science fiction are both interested in a future political system, political scientists, unlike fiction writers, are “limited by their preoccupation with the past and the present” (p. 8).

Speculative fiction is particularly useful for abolitionist research because it allows us to remove ourselves from the current capitalist and exploitive penal solutions to social and economic issues, creating space instead to imagine new, more egalitarian worlds. Abolitionists benefit from the freedom to imagine alternatives in a way that does not restrict or dampen their potential. Prisons bear little relationship to fluctuations in crime yet many people, or rather, most people, consider prisons intrinsic to society. Speculative fiction allows us to world build without prisons, to demonstrate that there are other, more just, more economically smart, more humane alternatives and solutions to the problems highlighted throughout this article. In combination with the freedom to explore beyond the past and present, speculative fiction offers a chance to translate these ideas into digestible and meaningful stories for audiences beyond academics, thus, making it a powerful tool for abolitionist researchers who face the daunting tasks of both addressing current penal realities and offering an alternative future.

Speculative fiction, in this article, will be used to explore new, more hopeful alternative ideas to current penal solutions. These alternatives strive to directly tackle some of the issues highlighted throughout this article. Imagining these alternatives through speculative fiction relieves the author of the tendency to rely on existing criminal justice systems that have historically over-criminalised LGBTQ + communities. Instead, the research asks, ‘How can we challenge traditional penal approaches to LGBTQ + issues, fostering alternative perspectives and solutions?’. Below, I use speculative fiction to explore it potential for fostering alternative perspectives and solutions. I present a series of flash fiction stories that highlight a range of queer ‘real’ utopian alternatives to prison. This is by no means an exhaustive list of alternatives, nor does it serve as a ‘blueprint’ for all the changes necessary to abolishing prisons. Rather, this article explores some of the potentialities of queer abolition real utopias and encourages queer criminology to focus and centre future works on abolition.

I have written three pieces of flash fiction to demonstrate the possibilities of queer abolition real utopias. These stories have been written and inspired by elements of my PhD research that explores LGBTQ + prisoners experiences in the UK. This was a multifaceted process of thinking through and reflecting on some of the core challenges facing LGBTQ + people in conflict with the law, ensuring that there was sufficient academic literature to situate the story in, and allowing myself to write creatively and imaginatively about alternative approaches to these issues, while also balancing the key features of a ‘real utopia’. To begin with, I wrote a ‘raw’ version of each of these stories that concerned itself only with the challenge and the alternative solution. I then refined these stories by returning to the literature which helped contextualise the key issues. Furthermore, to help balance some of those elements of utopianism and creativity with a more pragmatic approach, I draw inspiration from a wide range of existing alternative approaches and programmes, including penal mediation, justice reinvestment, the Audre Lorde Project, Steppingstone San Diego, and Akt. This allows the non-penal, community-centred approaches I bring into the stories to have elements of reality and true possibility attached to them.

The first piece of writing is titled *Finding Harmony House.* The protagonist is a young trans woman who ran away from home at a young age. Her life on the streets and later in a youth detention centred are discussed briefly. She is offered a chance to stay at Harmony House, an LGBTQ + shelter run by two women. Despite her reservations and existing assumptions about shelters, she accepts the offer and much to her surprise, makes great progress while staying at Harmony House. The second piece of writing is written as a transcript of a community forum held in the East end of Glasgow. The community forum focusses on handling conflict without criminal law and building conversations and toolkits for community-led approaches to tackle violence. The writing offers a short peek at the individuals in attendance at the meeting and why they think community-led approaches rather than police involvement are important for their community. The third and final piece of writing is about a young mother, Fiona, who had two children with her ex-partner, before coming to terms with her sexuality and coming out. Her ex-partner and the father of the children cuts all contact, leaving Fiona struggling to make ends meet. We are then introduced to Judy, the family worker, who is there to support our protagonist. Judy is patient, gracious and caring toward Fiona, offering her support Fiona could never have dreamed of. Fiona is delighted to learn about Leuchars Lodge, a weekend retreat organised by Judy for her and the children. The flash fiction writing is followed by a discussion that contextualises the story and situates it within the relevant academic literature.

## Imagining Queer Real Utopias

### Finding Harmony House

I ran away when I was 15. My stepdad was abusing me and my mum. I had hoped she would run with me, but she couldn’t. It started when I was 7, calling me a poof and pushing me around. When I came out it got worse. I never exactly told him I was trans, but I think deep down he knew. I finally had the courage to leave the day after my 15th birthday. I met others who had run away, some of them a lot older than I was, but they kept me safe on the streets at first. I ended up in Juvie at 16 and spent some time in and out of there. When I came out I had nowhere to go, so it was easier to just stay put. I kicked off on purpose and got myself an extra few nights every time. The last time one of the staff members sat me down and told me about Harmony House. She asked if I would be interested; they were hoping eventually places like Harmony House would prevent the need for Juvie at all. At first, I was a bit hesitant. My friend Amanda had told me some proper horror stories about the last shelter she was in. I was assured Harmony House wasn’t like that. I knew I had to trust what she was saying—I’m almost 20, if I keep going this way it’ll not be juvie next time. I was told Harmony House was for unhoused LGBTQ + youth and that I’d get my own room. That was all I really knew. When I turned up, I was surprised by how nice everyone was. It wasn’t like Amanda’s place. The two women who run it are married, one of them works in education and the other one had crawled her way out of the same cycle as me to work for different charities before opening Harmony House. There are currently 16 of us living here. Ellen is the newest recruit. She’s been detoxing since she got here, we are all really proud of her. The withdrawals look horrible—shaking; throwing up; no sleep. It’ll be worth it. She hasn’t been clean since she was 13, she used to get through the nights and the men she didn’t want to sleep with. Kevin is also quite new, his parents chucked him out the house last year and he’s been on friends’ sofas since then. He makes us all laugh with his constant complaints—I’m pretty sure he’s just joking most of the time. Last night he ordered Pizza and spent ten minutes complaining about there not being enough cheese. He still ate it all of course. The best thing about being here is that now because I’ve got an address, I’ve managed to get a job at B&M down the road. The pay isn’t great, but I like the people—and the 20% discount—enough to stay. I’m hoping I can go to college next year and sit some exams.

### Glasgow East End Community Forum

Moderator: Hi everyone. Thank you so much for being here today. We really appreciate your time and dedication to this work. We are here to discuss what handling conflict without criminal law means. Specifically, we are interested in understanding why and how LGBTQ + populations might do this. The aim of doing this together is to gain a better understanding of what conflict without law means, why LGBTQ + folks might be particularly interested in this and ultimately how we can help foster the use of community-led approaches to violence in Glasgow. To start us off, I want to firstly ask the group what brought you here? Please feel free to introduce yourselves and share a little bit about why you’re here with the group. Maybe we can just go round in a circle from here?

Stanley: Hi folks. I’m Stanley (He/Him). Born and raised in the East end of Glasgow. Growing up, there were always police around, I had a lot of mates basically on first name basis with the officers in that area. We were always polite to them, and for the most part, them to us. But the older I’ve gotten, the more resentful I’ve grown toward the police. I’ve been battered and witnessed other people being battered by them, I’ve seen mates arrested when really they’re the victims. So, when I saw the poster for this, I wanted to come and say my piece and find out more about what alternatives we have to calling the police, to see how other people handle conflicts.

Moderator: Thank you for sharing that, Stanley.

Benji: Hiya. I’m Benji. I’d say my experience is very similar to Stanley’s. I’ve had too many bad experiences with the police to want to pick up the phone and call them when I need help. If I see two guys being mocked or antagonised in the street for holding hands, I try to break it up and warn them to get lost before I call the police. It gives people an opportunity to cool off before someone else comes and puts their hands on them. Most of the time I don’t actually call the police—it’s not like they’ll really do anything. So, I want to learn how to handle these situations better. I want to be able to say to these guys “Enough. What’s your problem with two guys holding hands? Let’s actually talk about what’s going on here instead of throwing fists at each other”. There must be another way about this.

Moderator: Absolutely. Thank you Benji.

Kim: I feel like my relationship to the police is a bit different. Oh sorry. Hi. I’m Kim. Also, a Glasgow local. I wouldn’t say I’ve had bad experiences with the police necessarily. I’ve got family that are in the police. I do think, however, that their time and resources could be better spent on other things and not like “petty crime” I guess. I don’t feel like every time there’s something wrong, we need to call them. They’re already strapped for time. I’m interested in how we can, as a community, as an entire city maybe, figure out a way to rely on the police less and handle things ourselves a bit better.

Moderator: Mm, yes. Thank you, Kim.

Melanie: Hi, I’m Melanie, I just want to firstly say that I totally get you where you are coming from Kim, I understand this idea that all police are bad can be frustrating. But I think the fact that there are so many LGBTQ + people on the poverty line, dealing with difficult circumstances that increase the chances of them interacting with the police and therefore potentially getting arrested make this a much bigger discussion than bad cop/good cop can capture. We need to find a different way to deal with things. I’m here to figure out what part I can play in keeping other LGBTQ + people off the streets and out of prison.

Kim: I’m with you there Melanie. I think together we can surely come up with something.

Moderator: Absolutely. Over the course of the next few hours, we hope to do exactly that. So, thank you all again for being here. I’m going to get us started by thinking about who we think should be involved in what we call ‘community-based response’? Who, do you consider, are the community?

### Our Long Weekend at Leuchars Lodge

The kids are back to school in two weeks and I’ve still to get their school uniforms sorted. Ethan is growing rapidly and will need new shoes soon too. Calum’s music teacher has gotten him interested in playing the drums, so I need to sort the forms and money out for that as well. How do I explain the washing machine has just broken and that’s going to cost a couple hundred to replace too? Or more importantly, how do I explain that their ‘hero’ of a father won’t help them because their mother is gay and doesn’t want him? It just feels like everything is hitting a boiling point. I don’t want to fall back into old patterns. Judy, the family worker, is due a visit later today and I don’t want her to think I’m failing. The house is clean, I’ve not been drinking as much, I’ve got a few paint jobs lined up.

Judy arrives and I make her a cup of tea, 1 sugar, too much milk—just the way I know she likes it. I potter about, bringing her different biscuit options, asking how she’s been. “You’re nervous—why?” she asks. I realise I’m stalling. I don’t want to know what she has to say. “You’re doing well Fiona, I can see how hard you’ve been working”, she says. Thank God, I think. I take a seat and thank her. I tell her about the paint jobs when the boys go back to school, I tell her I haven’t been drinking as much. She knows. She asks if Dennis has been in touch—no, he hasn’t. I tell her about the money situation, that I need a new washing machine, that the boys need their school uniforms, that Calum is starting the drums. He refuses to help. She tells me about a grant I can apply for. She pulls out some forms and tells me that it will be ok. I cry with relief. “I’ve got even better news Fiona”. “What?” I ask. “How would you and the boys like to go away for a few days, there’s a place near St Andrews called Leuchars Lodge. It’s for families working through difficult times. It’s there to give you a break and allow you to spend some quality time with the boys. We can arrange for you to go before the schools go back. It won’t be a problem”. There are a few rules at Leuchars Lodge, no drinking, no smoking etc. I can do that. We get picked up and dropped off. 3 nights, just us. There’s kayaking and forest walks you can do. Judy grins ear to ear as I accept the offer. She squeezes my shoulder, “Keep up the good work Fiona”.

## Discussion

### ‘Finding Harmony House’ Discussion

Twenty-four percent of homeless young people identify as LGBTQ + (Akt, 2021), meaning LGBTQ + people are overrepresented in this demographic. Durso and Gates (2012) and Choi et al., (2015) suggest that while non-LGBT issues are sometimes cited as the primary reason for homelessness among LGBTQ + youth, the most common reason for LGBTQ + youth leaving home or being kicked out of their home is related to their parents’ rejection of their gender identity and/or sexuality. In fact, 77% of surveyed LGBTQ + youth in the UK believe that coming out to their parents was the main factor for becoming homeless (Akt, 2021). Indeed, once homeless, LGBTQ + youth are more likely to engage in ‘risky behaviours’ that detrimentally affect their physical and mental health; this might include risky survival strategies such as casual and/or unprotected sex as well as more illicit drug use (Cochran et al., 2002; Corliss et al., 2011). Less than half (44%) of surveyed LGBQ + youth were aware of any housing support available to them, and less than one third (35%) contacted their local authority for support (akt, 2021). As such, it is clear there is a breakdown between LGBTQ + young people and local authorities/service providers in this area. There is gap in providing LGBTQ + young people the appropriate support and care required to allow a path without involvement from the criminal justice system. Importantly, there is also work to highlight the negative experiences LGBTQ + people experience when trying to access shelters (Coolhart and Brown, 2017; Castaldelli-Maia et al., 2022). The writing about Harmony House demonstrates the importance of access to safe spaces for our protagonist who represents many LGBTQ + young people today who have had to leave their homes. Harmony House is introduced as a potential alternative for young people who are facing homelessness and engaged in risky behaviours.

### ‘Glasgow East End Community Forum’ Discussion

Doug Meyer (2014) has argued that the increase in hate crime laws has only reinforced neoliberalism by expanding police and prosecutorial power. Despite the goal of reducing prejudice and discrimination, hate crime laws have negative consequences that outweigh the potential benefits—for example, more police power, harsher sentences, and ultimately more incarceration. Furthermore, Spade and Wilse (2015**)** argue that “hate crime activism disappears the interests of poor and non-white communities” (p. 44). In other words, when a white person reports a crime, black and other ethnic minorities are disproportionately punished. When it ignores racism, mainstream gay rights advocacy—which typically advocates for more hate crime legislations—accepts the status quo and asks for acceptance from the current social circumstance rather than challenging this “neutrality”. Indeed, given that the police have frequently administered anti-queer violence rather than preventing it, Meyer suggests that a progressive and radical queer critique should, rather, resist efforts to expand criminalisation and consider alternative means of reducing and preventing violence. The community forum is introduced as a potential means to engage the local community in community-based efforts to reduce and prevent violence. The forum is written as a transcript, introducing the many voices of those who volunteered to take part in the discussion. The writing demonstrates the importance and potential of local community organising efforts to reduce police involvement and create positive community links.

### ‘Our Long Weekend at Leuchars Lodge’ Discussion

Despite the myth of gay affluence, there is a growing body of literature to suggest that LGBTQ + people are less financially secure than their heterosexual, cisgender counterparts (DeFilippis, 2016). Historically, LGBTQ + individuals have faced outright bans in certain employment sectors, including teaching and the military (Badgett et al., 2021). Still today, Galupo and Resnick (2016) and Bayrakdar and King (2021) document the negative experiences of LGBTQ + individuals face in the workplace, and the impact this has on their job satisfaction. This leads to an overall wage and income difference among LGBTQ + individuals and their heterosexual counterparts (Valfort, 2017; Burn 2019; Martell, 2019). Leuchars’s Lodge tells the story of Fiona, a working single mother whose husband abandoned the family after she came out as gay. Fiona struggles with job security and lack of familial support as she navigates motherhood alone. Relatedly, DeChants et al (2022) discuss family rejection as the first system of support to collapse for LGBTQ + people. Rejection might include withdrawal of support and affection as well as through physical and psychologically harmful behaviours (Rohner et al., 2004**)**. The overwhelmingly negative impact of family rejection on LGBTQ + people has been well documented (Klein and Golub 2016; Drydakis, 2021). While Fiona has been rejected by her ex-husband and seems to receive little to no support from other family members, she is embraced and supported by a family worker, Judy. This writing encourages us to recognise LGBTQ + poverty as a social issue, and to consider new ways to support people facing these challenges. Fiona, had she not had the support of the family worker and the subsequent moments of respite provided to her, could have turned to more risky behaviours to cope with the challenges she is facing (Yadegarfard, 2020; Klein and Golub, 2016).

### Broader Discussion

While all three stories centre one or multiple characters’ unique and individual social and economic issues, these issues are pertinent among the wider LGBTQ + community, and the underlying themes of the stories share many commonalities. Firstly, the stories demonstrate the complex individual circumstances that can lead LGBTQ + people to become in contact with the police, and therefore in contact with the wider criminal justice system. In *Finding Harmony House*, the protagonist grew up in an abusive household and faced rejection from their stepfather for their identity. This leads to our young protagonist leaving home and trying to ‘find their own way’. In the *Glasgow East End Community Forum,* we hear from multiple characters about their experience with the police, with some citing their distrust stemming from growing up in an area with a large police presence. Finally, in *Our Long Weekend at Leuchars Lodge*, we hear about the struggle of a mother with two children. Fiona, the protagonist, has faced family rejection, alcohol dependency and in turn, struggled with maintaining employment. Her family are considered vulnerable and are in close contact with local authorities. In all three circumstances, we can see the trajectory of contact with local authorities to contact with the wider system. These stories present a crossroads for our characters. In many instances, the circumstances of these characters’ lives could and would indeed lead to contact with the wider criminal justice system. However, the story turns, and we find ourselves with three examples of non-penal, community-centred, and care-centred approaches. Our young protagonist in *Finding Harmony House* is offered a supportive alternative to prison; in the *Community Forum*, we see the characters coming together to talk about the police and indeed reframe their frustrations into community alternatives, and in *Our Long Weekend at Leuchars’s Lodge*, we see Fiona receive the support she needs to maintain her family life and mental well-being.

These stories aim to encourage us to think more deeply about complex and individual circumstances and reflect on the structural and systemic nuances at play in their stories, and how these in combination are more likely to lead LGBTQ + people into contact with the wider criminal justice system. These stories, in turn, aim to inspire people to think differently about how we can manage and support people through community efforts. These stories can be used in multiple other ways; for example, to encourage others to reflect on non-penal approaches or to encourage us to consider the impact of other social and economic issues. This could be a helpful classroom exercise on imagining alternatives for sociology or social work students. I would encourage everyone to reflect on their own individual circumstances and the support they might like to receive, in order to ground these alternatives in lived experience and humanity.

## Conclusion

This article has explored the potential for queer abolition ‘real’ utopias using speculative fiction as a method. Speculative fiction as a method allows researchers and writers to remove themselves from current penal solutions and think creatively and beyond the existing systems of control to imagine new, more just paths forward. The writing above does not demonstrate all the possibilities, or indeed the challenges involved in these alternatives. However, the writing does demonstrate that alternatives are possible, tackling both the current social and penal realities of LGBTQ + people as well as centring new alternatives.

The flash pieces of fiction demonstrate queer abolition real utopias—they are specific to the queer community; they each have an element of utopianism, but they are also real and plausible alternatives that would help reduce the need for current penal solutions. In *Finding Harmony House,* the central idea rests on the use of LGBTQ + youth shelters rather than juvenile detention centres. In the *Glasgow East End Community Forum,* the forum itself is the central idea of the story. The story demonstrates the simplicity of bringing people together to discuss community-based solutions to violence, in the hopes of forging community and gaining a better understanding of the experiences of LGBTQ + people and the police. And lastly, *Our Long Weekend at Leuchars Lodge* centres the importance of respite and family support amidst financial difficulties and family rejection. The creative writing throughout this article attempts to balance the central idea, which is an alternative to the current criminal justice solutions, with authentic and plausible characters facing issues which many LGBTQ + people today face. In doing so, speculative fiction is used to carefully remove the current penal solutions that are so engrained in society and indeed in our current imaginations and encourages us to consider new directions.

Overall, the article demonstrates what speculative fiction could offer criminology and the more specific tasks of theorising prison alternatives. The article contributes to the range of literature that is encouraging social scientists to be more creative in their writing and dissemination. While certainly, speculative fiction can be cathartic and even fun for researchers, it also holds the crucial and critical power to encourage us in new directions that we may not otherwise find. Thus, making it a powerful tool for abolitionist researchers interested in theorising beyond the past and present realities. Furthermore, this article is a call for queer criminology and those engaged in prisons research to engage in more expansive and radical work that both attends to the current penal and social realities of marginalised communities, but that also pushes boundaries and seeks a brighter, more hopeful future. Queer criminology should be engaging in this kind of work, it should be consistently pushing boundaries and striving, if not demanding, more of criminology.

## References

[CR1] Akt, (2021) The LGBTQ+ Homelessness Report. Available online at: https://www.akt.org.uk/wp-content/uploads/2023/07/akt-thelgbtqyouthhomelessnessreport2021.pdf

[CR2] Badgett, L., Carptenter, C., and Sansone, D., (2021) LGBTQ Economics. *Journal of Economic Perspectives,* 35(2)

[CR3] Ball, M., (2016) Queer criminology as activism. *Critical Criminology*, 24(4), 10.1007/s10612-016-9329-4

[CR4] Bayrakdar, S., and King, A., (2021) Job Satisfaction and Sexual Orientation in Britain. *Work, Employment and Society,* 36(1) 10.1177/0950017020980997

[CR5] Burn, I., (2019) The relationship between prejudice and wage penalties for gay men in the United States. *IRL Review,* 73(3) 10.1177/0019793919864891

[CR6] Butler, O. E., (1993) *Parable of the Sower*. Headline.

[CR7] Carr, N., McAlister, S. & Serisier, Tanya. (2016) Out on the inside: the rights, experiences and needs of LGBT people in prison. Project Report. Irish Penal Reform Trust, Ireland. Available at https://www.iprt.ie/site/assets/files/6369/iprt_out_on_the_inside_2016_embargo_to_1030_feb_02_2016.pdf

[CR8] Castaldelli-Maiai, J, M., and Centriglio, A., and Bhugra, D., (2022) *Homelessness and Mental Health*. Oxford: Oxford University Press.

[CR9] Choi, S. K., Wilson, B, Shelton, J. (2015) Serving Our Youth 2015: The Needs and Experiences of Lesbian, Gay, Bisexual, Transgender, and Questioning Youth Experiencing Homelessness. *UCLA*: *The Williams Institute*. Retrieved from https://escholarship.org/uc/item/1pd9886n

[CR10] Cochran, B., Stewart, A., Ginzler, J., and Cauce, A., (2002) Challenges Faced by Homeless Sexual Minorities: comparison of lesbian, bisexual, and transgender homeless adolescents with their heterosexual counterparts. *American Journal of Public Health,* 92(1) 10.2105/AJPH.92.5.773 10.2105/ajph.92.5.773 PMC1447160 11988446

[CR11] Cohen, S. (1990) Intellectual scepticism and political commitment: the case of radical criminology In Walton, P and Young, J (eds) The New Criminology Revisited. London: Macmillan Press.

[CR12] Colledge, L., Hickson, F., Reid, D., and Weatherburn, P., (2015) Poorer mental health in UK bisexual women than lesbians: evidence from the UK 2007 Stonewall Women’s Health Survey. *Journal of Public Health,* 37(3), 10.1093/pubmed/fdu105 10.1093/pubmed/fdu105 25586100

[CR13] Coolhart, D., Brown, M., (2017) The need for safe spaces: Exploring the experiences of homeless LGBTQ youth in shelters. *Children and Youth Services Review,* 82(1), 10.1016/j.childyouth.2017.09.021

[CR14] Copson L., Boukli A. (2020) Queer utopias and queer criminology. *Criminology & Criminal Justice*, 20(5), 10.1177/1748895820932210

[CR15] Corliss, H., Goodenow, C., Nichols, L and Austin, B., (2011) High burden of homelessness among sexual-minority adolescents: Findings from a representative Massachusetts high school. *American Journal of Public Health,* 101(1) 10.2105/AJPH.2011.300155 10.2105/AJPH.2011.300155 PMC3154237 21778481

[CR16] D’Avolio, M., Cavalcanti, R. P., and Dadusc, D., (2023) Anti-carceral feminism: abolitionist conversations on gender-based violence In Chatterjee and Lee (eds) Plural Feminisms: Navigating Resistance as Everyday Praxis. London: Bloomsbury Publishing.

[CR17] Davis, A., (2003) Are Prisons Obsolete? Seven Stories Press.

[CR18] DeChants, J., Shelton, J., Anyon, Y., and Bender, K., (2022) “It kinda breaks my heart: LGBTQ young adults’ responses to family rejection. *Family Relations,* 71(3) 10.1111/fare.12638

[CR19] DeFillippis, J. N., (2016). “What About the Rest of Us?” An Overview of LGBT Poverty Issues and a Call to Action. *Journal of Progressive Human Services,* 27(3) 10.1080/10428232.2016.1198673

[CR20] Donohue, G., McCan, E., and Brown, M., (2021) Views and Experiences of LGBTQ+ People in Prison Regarding their Psychosocial Needs: A Systematic Review of the Qualitative Research Evidence. *Int. J. Environ, Res. Public Health,* 18(17) 10.3390/ijerph18179335 PMC8430972 34501930

[CR21] Drydakis, N., (2021) Social Rejection, family acceptance, economic recession, and physical and mental health of sexual minorities. *Sexuality Research and Social Policy,* 19(1) 10.1007/s13178-021-00640-4

[CR22] Durso, L. E., and Gates, G. J., (2012) Serving Our Youth: Findings from a National Survey of Services Providers Working with Lesbian, Gay, Bisexual and Transgender Youth Who Are Homeless or At Risk of Becoming Homeless. *UCLA: The Williams Institute*. Retrieved from https://escholarship.org/uc/item/80x75033

[CR23] ElShafie, S. (2018) Making Science Meaningful for Broad Audiences through Stories. *Integrative & Comparative Biology,* 58(6) 10.1093/icb/icy103 10.1093/icb/icy103 30059998

[CR24] Fernandes, F., Kaufmann, B. & Kaufmann, K. (2020) LGBT+ People in Prisons: Experiences in England and Scotland. Full Report. University of Dundee, UK. Available at: https://discovery.dundee.ac.uk/files/56478375/LGBT_People_in_Prisons_Full_Report_09_FEB_21_WEB.pdf

[CR25] Galupo, A., and Resnick, C., (2016) Experiences of LGBT microaggressions in the workplace: implications for policy In: Köllen, T. (eds) *Sexual Orientation and Transgender Issues in Organizations*. Springer, Cham.

[CR26] Gerlach N, and Hamilton S, N., (2003) “Introduction: A History of Science Fiction.” *Science Fiction Studies* 30(2)

[CR27] Giddens, A., (1994) *Beyond Left and Right*. Cambridge: Polity Press.

[CR770] Greenberg, M., and Warrick, P., (1974) *Political Science Fiction: An Introduction*. Prentice-Hall.

[CR28] Government Equalities Office., (2018) National LGBT Survey. Summary Report. Available at: https://assets.publishing.service.gov.uk/media/5b3cb6b6ed915d39fd5f14df/GEO-LGBT-Survey-Report.pdf

[CR29] Hobson E. K. (2016). *Lavender and red: Liberation and solidarity in the gay and lesbian left*. University of California Press.

[CR30] Houlden, S., and Velestianos, G., (2023) Impossible dreaming: On speculative education fiction and hopeful learning futures. *Postdigital Science and Education,* 5(1), 10.1007/s42438-022-00348-7 10.1007/s42438-022-00348-7 PMC9589552 40479381

[CR31] Jorm, A.F., Korten, A. E., Rodgers ,B., Jacomb, P. A.,& Christensen, H. (2001) Sexual orientation and mental health: Results from a community survey of young and middle-aged adults. *British Journal of Psychiatry*, 180(5), 10.1192/bjp.180.5.423 10.1192/bjp.180.5.423 11983639

[CR32] Kãrklina, A., (2021) Abolitionist Futures: Black Cultural Imagination at the End of the World. Available at: https://dukespace.lib.duke.edu/server/api/core/bitstreams/c23a92b8-45b7-4b30-943f-6106f1e13924/content

[CR33] Kim, M. E., (2018) From carceral feminism to transformative justice: Women-of-colour feminism and alternatives to incarceration. *Journal of ethnic and cultural diversity in social work,* 27(3) 10.1080/15313204.2018.1474827

[CR34] Kithcin, R., (2013) Engaging public: writing as praxis. *Cultural Geographies,* 21(1) 10.1177/1474474012462535

[CR35] Klein, A., and Golub, A., (2016) Family rejection as a predicator of suicide attempts and substance misuse among transgender and gender nonconforming adults. *LGBT Health*, 3(3), 10.1089/lgbt.2015.0111 10.1089/lgbt.2015.0111 27046450

[CR36] Lamusse, T., (2022) Feminist prison abolition In Gibbs, A., and Gilmour, F (eds) *Women, Crime and Justice in Context*. New York: Routledge.

[CR37] Le Guin, U. K., (1974) *The Dispossessed*. Orion.

[CR38] Martell, M., (2019). Age and the new lesbian earnings penalty. *International Journal of Manpower,* 41(6), 10.1108/IJM-10-2018-0322

[CR39] Maycock, M. (2022) The Transgender Pains of Imprisonment. European Journal of Criminology, 19(6), 10.1177/1477370820984488

[CR40] Meyer, D., (2014) *Violence against Queer People: Race, Class, Gender, and the Persistence of Anti-LGBT Discrimination*. Rutgers University Press.

[CR41] Peterson, D., and Panfil, V. R. (2013) *Handbook of LGBT Communities, Crime, and Justice*. New York: Springer.

[CR42] Prison Reform Trust., (2021). Bromley Briefings Prison Fact file. Winter 2021. Available at: https://prisonreformtrust.org.uk/wp-content/uploads/2022/01/Bromley_Briefings_winter_2021.pdf

[CR43] Richardson, L. (2000) “Writing: A Method of Inquiry.” In *Handbook of Qualitative Research* (2nd ed.), edited by Denzin Norman, Lincoln Yvonna S. Thousand Oaks, CA: Sage

[CR44] Rinehart, R. (1998) “Fictional Methods in Ethnography: Believability, Specks of Glass, and Chekhov.” *Qualitative Inquiry* 4(2), 10.1177/107780049800400204

[CR45] Rodgers, J., Asquit, N., and Dwyer, A., (2017) Cisnormativity, criminalisation, vulnerability: Transgender people in prisons. Tasmanian Institute of Law Enforcement Studies, 12(1)

[CR46] Rodriguez S. M. (2021). Queer abolitionist alternatives to criminalising hate violence. In Coyle M. J., Scott D. (Eds.), *The Routledge international handbook of penal abolition* (pp. 190–200). Routledge.

[CR47] Rohner, RP., Khaleque, A., and Cournoyer, DE. (2004) Cross-national perspectives on parental acceptance-rejection theory. *Marriage and Family Review,* 35(3–4) 10.1300/J002v35n03_06

[CR48] Russel, E and Carlton, B., (2013) Pathways, race and gender responsive reform: Through an abolitionist lens. *Theoretical Criminology,* 17(4) 10.1177/1362480613497777

[CR49] Scott, D., (2013) Visualising an abolitionist real utopia: principles, policy, and praxis In Malloch and Munro (eds) *Crime, Critique and Utopia.* Palgrave Macmillan.

[CR50] Spade, D., and Wilse, C., (2015). Confronting the limits of gay hate crimes activism: A radical critique. CHICANO-LATINO L. Rev. 38

[CR51] Stanley E. A. (2011) Fugitive flesh: Gender self-determination, queer abolition, and trans resistance. In Stanley E. A., Smith N. (Eds.), *Captive genders: Trans embodiment and the prison industrial complex* (pp. 7–17). AK Press

[CR52] Stonewall., (2018) LGBT in Britain – Trans Report 2018. Available at: https://www.stonewall.org.uk/system/files/lgbt_in_britain_-_trans_report_final.pdf

[CR53] Taylor, C., (2018) Anti-carceral feminism and sexual assault – A Defense. *Social Philosophy Today*, Online First, DOI: 10.5840/socphiltoday201862656

[CR54] Terwiel, A., (2019) What is Carceral Feminism? *Political Theory*, 48(4), 10.1177/0090591719889946

[CR55] Thorne, S., (2021) Through Critique and Beyond: Speculative Fiction as a Tool of Critical Pedagogy. *Master’s Projects and Capstones*. 1288. https://repository.usfca.edu/capstone/1288

[CR56] Valfort, M., (2017) LGBTI in OECD Countries: A Review. OECD Working Paper. 10.1787/d5d49711-en

[CR57] Velasquez-Potts, M., (2021) Abolitionist Generosities: On Hunger Striking as Queer Refusal. *QED,* 8(3) 10.2307/48657434

[CR58] Vogler, S., and Rosales, R., (2022) Classification and Coercion: The Gendered Punishment of Transgender Women in Immigration Detention. *Social Problems*, 70(3) 10.1093/socpro/spac022

[CR59] Walker, A., Peterson, A., Wodda, A, and Stephens, A., (2022) Why Don’t We Center Abolition in Queer Criminology? *Crime and Delinquency,* 0(0), 10.1177/00111287221134595

[CR60] Walters, R., Antojado, D., and Bartels, L., (2024) LGBT people in prison in Australia and human rights: A critical reflection. *Alternative Law Journal*, 49(1) 10.1177/1037969X241231007

[CR61] Wright, E. O. (2010) *Envisioning Real Utopias*. London: Verso.

[CR62] Yadegarfard, M. (2020) ‘Biopsychosocial Predictors of Risky Sexual Behaviours Among the Gay Men in the UK. Volume 1 of 2’. PhD thesis. University of Bedfordshire.

